# A Seventeen-Year Epidemiological Surveillance Study of *Borrelia burgdorferi* Infections in Two Provinces of Northern Spain

**DOI:** 10.3390/ijerph110201661

**Published:** 2014-01-30

**Authors:** Lourdes Lledó, María Isabel Gegúndez, Consuelo Giménez-Pardo, Rufino Álamo, Pedro Fernández-Soto, María Sofia Nuncio, José Vicente Saz

**Affiliations:** 1 Department of Biomedicine and Biotecnology, Alcalá University, 28871 Alcalá de Henares, Spain; E-Mails: isabel.gegundez@uah.es (M.I.G.); consuelo.gimenez@uah.es (C.G.-P.); josev.saz@uah.es (J.V.S.); 2 Territorial Health Service and Social Welfare of the Junta de Castilla y León, 47003 Valladolid, Spain; E-Mail: alasanru@jycl.es; 3 Department of Parasitology, Salamanca University, 37008 Salamanca, Spain; E-Mail: pfsoto@usal.es; 4 Centre for Vectors and Infectious Diseases Research, Instituto Nacional de Saúde Dr. Ricardo Jorge (National Institute of Health), 2965-575 Aguas de Moura, Portugal; E-Mail: sofia.nuncio@insa.min-saude.pt

**Keywords:** Lyme disease, epidemiology, public health

## Abstract

This paper reports a 17-year seroepidemiological surveillance study of *Borrelia burgdorferi* infection, performed with the aim of improving our knowledge of the epidemiology of this pathogen. Serum samples (1,179) from patients (623, stratified with respect to age, sex, season, area of residence and occupation) bitten by ticks in two regions of northern Spain were IFA-tested for *B. burgdorferi* antibodies. Positive results were confirmed by western blotting. Antibodies specific for *B. burgdorferi* were found in 13.3% of the patients; 7.8% were IgM positive, 9.6% were IgG positive, and 4.33% were both IgM and IgG positive. Five species of ticks were identified in the seropositive patients: *Dermacentor marginatus* (41.17% of such patients) *Dermacentor reticulatus* (11.76%), *Rhiphicephalus sanguineus* (17.64%), *Rhiphicephalus turanicus* (5.88%) and *Ixodes ricinus* (23.52%). *B. burgdorferi* DNA was sought by PCR in ticks when available. One tick, a *D. reticulatus male*, was found carrying the pathogen. The seroprevalence found was similar to the previously demonstrated in similar studies in Spain and other European countries.

## 1. Introduction

Arthropod-borne diseases are the most common zoonoses involving wildlife in the northern hemisphere, especially the Old World [1]. *Borrelia burgdorferi*, the causal agent of Lyme borreliosis (LB), is found worldwide and comprises at least 18 genospecies [2,3]. Discovered in 1982, *B. burgdorferi* is now considered an emerging (and possibly under-reported) pathogen that could become an important public health problem [4]. *Ixodes ricinus* is the tick that usually transmits this pathogen to humans in Europe [5].

In recent years there has been a substantial increase in interest in *B. burgdorferi* infection, and different epidemiological studies have been made in different parts of Spain [6,7,8,9]. Clinical cases have also been reported [10,11,12,13]. Thus far, the prevalence of LB has been studied in high-risk groups, in the general population, and in patients showing possible signs of infection [12,14,15,16,17]. Ongoing studies at the local level are required to construct more efficient risk prediction models. In response to this need the present work reports a 17-year epidemiological surveillance study of *B. burgdorferi* infection in two areas of northern Spain where this pathogen is known to exist.

## 2. Material and Methods

### 2.1. Study Area

This study was performed in the Province of Palencia (central coordinates 42°00'23" N, 4°31'45" W, mean altitude 749 m) and in a small area in the Province of Burgos (central coordinates 42º20'57.1" N, 3°41'4.7" W, mean altitude 856 m). The mean daily summer temperature in these areas is 36.2 °C, while the mean winter temperature is 7.5 °C. Both areas are mainly rural, but recreational activities have attracted non-residents to them in recent years. Animal husbandry is economically important in both areas, where the parasitisation of livestock by ticks is common. Both areas provide the conditions under which LB infections usually occur.

### 2.2. Serum Samples

Serum samples were collected from patients bitten by ticks - and therefore at risk of *B. burgdorferi* infection—over a 17-year period (May1996–May 2013). A total number of 1,179 serum samples from 623 patients (age range 3 months to 91 years) were available: 601 from the Palencia and 22 from the Burgos study areas. By sex 345 (55.3%) of these samples came from males and 278 from females (44.7%). All sera were collected from patients at primary healthcare centres. Blood was taken once (at first presentation) from 225 patients (36.1%), twice (at first presentation and 30 days later) from 240 patients (38.5%), and three times (at first presentation, 30 days later, and at 90 days) from 158 patients (25.3%). All serum samples were maintained at −20 °C until analysis. The following information was collected from all patients: age, sex, occupation, place of residence, symptomatology, contact with cattle, profession, and information regarding their tick bite.

All the patients gave their informed consent to be included in the study (all adult participants and from the parents or legal guardians of minors), in compliance with the ethical standards of the Human Experimentation Committee of the University of Alcalá de Henares and the Helsinki Declaration of 1964 (as revised in 2004). All patients that came to a health centre with fever were treated with antibiotics.

### 2.3. Immunofluorescence

**Figure 1:**
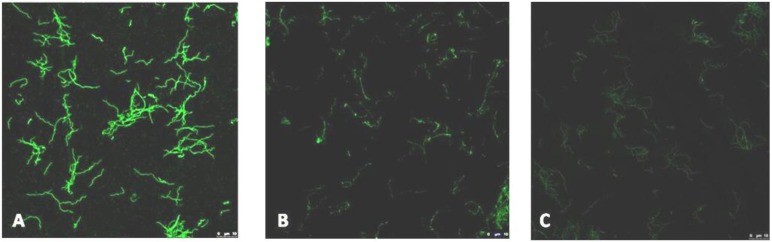
Typical pattern of *B. burgdorferi* IFA. (**A**) Positive sera; (**B**) Uncertain sera; (**C**) Negative control.

Sera were tested for *B. burgdorferi* antibodies by a home-made indirect immunofluorescence assay (IFA). The *Borrelia* employed as the antigen was *B. burgdorferi sensu stricto* (strain B31 ATCC 35210). These bacteria were propagated in Barbour Kelly medium and fixed on spot slides. The fluorescein-labelled conjugates used were rabbit anti-human IgG and IgM (Sigma, St Louis, MO, USA), diluted 1/128 in PBS. Briefly, two-fold dilutions of each serum sample were added to the antigen spots and incubated in a humidity chamber for 30 min at 37 °C. After washing, the conjugate was added to each sample. The slides were then incubated for 30 min, washed, and examined using a BH2 fluorescence microscope (10 × 40, Olympus, Tokyo, Japan). Positive and negative control sera were also examined. Sera showing a typical pattern of fluorescence at IgG titres of ≥1:256 and IgM titres of ≥1:32 were deemed positive (Figure 1). All positives sera were tested for infection by *Treponema pallidum* by the haemagglutination test (TPHA-BioMérieux, Marcy l´Etoile, France) to rule out syphilis.

### 2.4. Sodium Dodecyl Sulphate Polyacrylamide Gel Electrophoresis and Western Blotting

**Figure 2:**
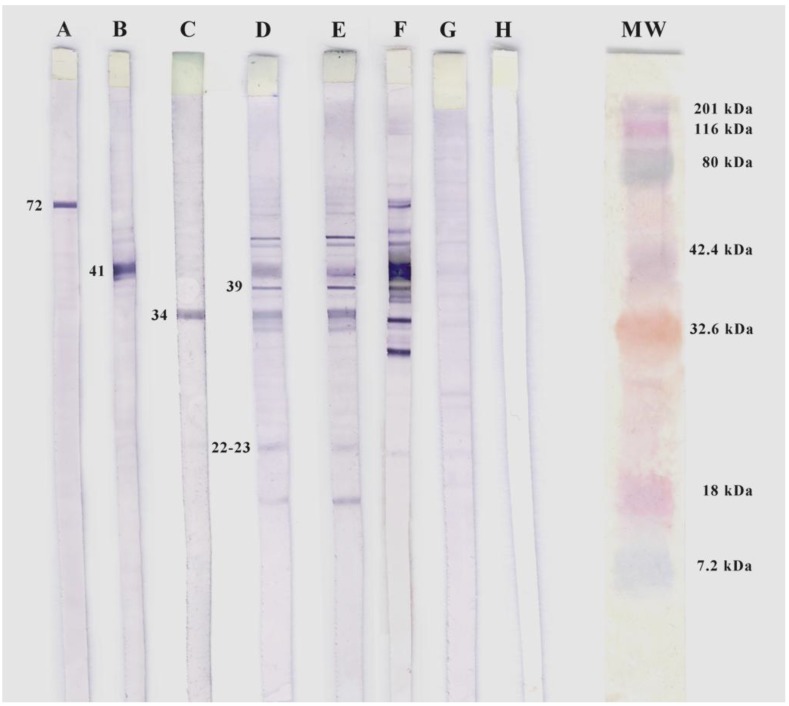
Western blotting of *B. burgdorferi*. A: MAb CB312 ; B: MAb H9724 ; C: MAb 84C; D and E: Sera positive patient for IgM (second and third samples respectively); F: Positive control; G: Negative control; H: white control (PBS); MW: molecular weight (BioRad).

All sera with an uncertain or positive IFA result were subjected to a home-made western blotting (WB) test. WB strips were prepared with sonicated *B. burgdorferi* (strain B31 ATCC 35210) cells [18]. Electrophoresis was performed at 125 V for 90 min and the separated proteins transferred to PDVF membranes. These membranes were then cut into strips, hydrated with methanol, and blocked with 5% gelatin (Bio-Rad, Hercules, CA, USA). Human serum, diluted 1:200 in TTBS 1% (Tris-NaCl, 1% gelatin, 0.05% Tween-20), was then added and incubated at 37 °C/h for 2 h. The positive controls used included at least three different monoclonal antibodies (diluted 1:000) from the following list: 11G1 (OspA), CB312 (P72), CB2 (OspB), CB1 (Flagelina) (kindly provided by Dr. Pedro Anda, Centro Nacional de Microbiología, Majadahonda, Madrid), 181.1 (93 kDa), H9724 (Fla, 41 kDa) and 84C (OspB, 34 kDa). A human serum positive for *B. burgdorferi* (supplied by Dr. Sofia Nuncio, Centro de Estudos de Vetores e Doenças Infecciosas (CEVDI), Instituto Nacional de Saúde, Aguas de Moura, Portugal), diluted 1:200, was used as a further positive control. A human serum negative for the bacterium, also diluted 1:200, was used as a negative control, and a strip with PBS as white control. After washing with TTBS 0.01% (TBS, 0.1% gelatine, 0.05% Tween-20) alkaline phosphatase-conjugated goat anti-human IgG, anti-human IgM or total IgG anti-mouse (Sigma) antibodies were added and the preparations incubated and washed as described above. Blots were visualized using nitroblue tetrazolium and 5-bromo-4-chloro-3-indoyl-phosphate (Sigma). Serum specimens were deemed positive if they reacted with at least five of the following IgG diagnostic bands: 18 kDa, 23 kDa (Ospc), 28 kDa, 30 kDa, 39 kDa (BmpA), 41 kDa, 25 kDa, 58 kDa (not GroEL), 66 kDa or 93 kDa, and at least two of the following IgM diagnostic bands: 23 kDa (OspC), 39 kDa (BMPa) or 41 kDA (Fla) (CDC Recommendations, 1995) (Figure 2).

### 2.5. Analysis of Ticks for Borrelia burgdorferi DNA

Ticks, collected from the patients whenever possible, were decontaminated by sequential washing in 45% alcohol, 30% alcohol, and ultrapure water. Each tick was then transferred to an individual glass vessel in which it was cut into pieces using a sterile blade, and DNA extracted in 500 mL of 5% Chelex-100 (Bio-Rad, Hercules, CA, USA) [19]. *B. burgdorferi* DNA was sought by amplification of the 5S–23S rRNA intergenic spacer [20]. To prevent DNA contamination and the carryover of amplified products, sterile tools were used at all times, and each step of the analysis (extracting DNA, preparing the reaction mixture, and amplifying and analysing the PCR product) was performed in a separate work area. Two negative controls (Milli-Q water and DNA from laboratory-reared, non-infected ticks), and two positive controls (DNA from *B. burgdorferi* strain Esp-1) were included in all PCR runs.

### 2.6. Statistical Analysis

Differences in epidemiological results were compared using the χ^2^ test (performed manually). Significance was set at *p* < 0.05.

## 3. Results

**Figure 3:**
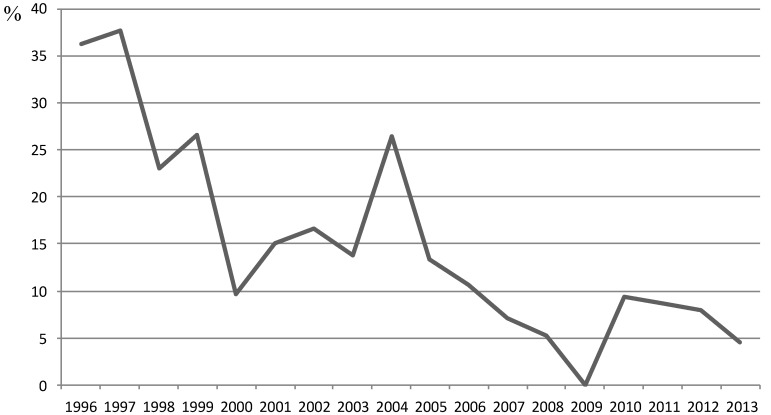
Distribution of seroprevalence by years.

Specific *B. burgdorferi* antibodies were detected in 83 (13.3%) patients (Figure 3); 43 males (12.46%) and 40 females (14.38%).

Seven positive sera came from people who lived in the Burgos region (31.8%) and 76 from people in the Province of Palencia (12.6%). All seropositive patients had contact with animals, and all age groups were represented (range 3–87 years). Table 1 shows there were no significant differences among age groups in terms of seropositivity.

**Table 1:** Seroprevalence in relation to age groups.

Age groups	Number (%)
0–10 years	8 (9.2)
11–20 years	10 (17.2)
21–30 years	5 (11.3)
31–40 years	14 (15.38)
41–50 years	19 (16.9)
51–60 years	7 (8.1)
61–70 years	10 (13.7)
71–80 years	8 (13.8)
81–90 years	2 (15.38)
91–more years	ND

ND: No done.

Table 2 shows there were no differences in seroprevalence in terms of patient occupation. No relationship was seen between seropositivity and province of residence. Most patients had been bitten in spring (315 people [50.56%]) or autumn (119 people [21.18%]).

**Table 2:** Seroprevalence in terms of patient occupation.

Patients occupation	Number (%)
Farmer	5 (7.9)
Livestock	3 (9.6)
Pastors	1 (7.7)
Construction workers	6 (25)
Administrative workers (clerks, lawyers, business)	4 (14.2)
Service sector (shopkeepers, waiters, cooks, mechanics, maintenance)	8 (25)
Veterinarians	1 (33.3)
Health (doctors and nurses)	2 (20%)
Houesewives	13 (12.6)
Retirees	10 (14.3)
Students	5 (7.35)
Unspecified profession (mostly children under five years)	25 (18.38)

After examining all patient sera, *i.e.*, including sera taken from patients up to three times, 49 (7.8%) were found to be IgM positive and 60 (9.6%) IgG positive; 27 patients (4.33%) had both IgM and IgG antibodies (Table 3 shows the distribution by years).

In those patients for whom more than one serum sample was available (*n* = 498), 8 (1.61%) showed rising IgG titres, five showed rising IgM titres (1.00%), and 4 (0.80%) showed increases in both IgG and IgM. Retro-seroconversion with respect to IgG occurred in six people (1.20%), and with respect to IgM in one person (0.20%). In both cases, seroconversion and retroconversion, occurred during the first years of the study, in particular between 1997 and 2004. Some patients remained completely asymptomatic while others showed influenza-like symptoms (fever, fatigue, headaches and joint pain).

**Table 3:** Seroprevalence in relation to years.

Years	Ig G prevalence (%)	IgM prevalence (%)	IgG+IgM prevalence (%)
1996	18.18	27.27	9.09
1997	15.55	35.55	15.55
1998	2.56	23.07	2.56
1999	20	13.33	6.66
2000	9.67	6.45	6.45
2001	15	10	10
2002	16.66	16.66	16.66
2003	13.79	3.44	3.44
2004	26.47	14.7	14.7
2005	10	6.66	3.33
2006	10.71	0	0
2007	7.14	0	0
2008	5.26	1.74	1.74
2009	0	0	0
2010	9.3	2.32	2.32
2011	6.89	3.44	1.74
2012	7.89	0	0
2013	4.54	0	0

Five species of ticks (17 ticks) were identified to have bitten the seropositive patients: *Dermacentor marginatus* (41.5%; 14.2% males and 85.71% females), *Dermacentor reticulatus* (11.76%; 50% males and 50% females), *Rhiphicephalus sanguineus* (17.64%; 66.6% males and 33.3% females), *Rhiphicephalus turanicus* (5.88%; 100% males) and *I. ricinus* (23.52%; 25% males and 75% females).

IgM antibodies expressed against *B. burgdorferi* were found in four patients who had been bitten by *D. marginatus*, in two patients bitten by *D. reticulatus*, in two patients bitten by *R. sanguineus*, and in one bitten by *I. ricinus*. A male of *D. reticulatus* was infected with *B. burgdorferi*. The tick was removed from the scalp of a 13-year-old woman in May 1997. The patient presented a week after the bite the presence of regional lymph nodes close to the area of the bite without fever. The patient had high titer of IgM and IgG antibodies against *B. burgdorferi*, but not seroconversion.

## 4. Discussion and Conclusions

Though Lyme disease is thought to have been present in Spain since 1977, its existence was not serologically confirmed until 1987 [10,21,22]. Since then, *B. burgdorferi* infections have been frequently recorded, with differences in prevalence from area to area, and its geographical distribution may be increasing as a result of climate change, changes in land uses, and evolving socioeconomic factors [10,23]. The present work is the longest-term (17 years) epidemiological surveillance study of *B. burgdorferi* ever performed in Spain. The seroprevalence of 13.3% detected in the present work falls within the range previously reported for Spain (0.3–14.8%) and other European countries (1.8%–19.68%) [24,25,26,27,28,29].

Ticks are abundant in rural areas, and it is reported that those persons whose work implied contact with animals in the rural setting are at higher risk of infection [30,31,32,33]. However, the prevalence of *B. burgdorferi* infection in high risk populations is not well known and seems to vary widely from area to area. In the present study, no significant differences in seroprevalence were seen between different occupation groups and by age, but others authors report studies that shown significant differences in seroprevalence data respect to occupation and/or age [32,34]. In the present work, similar seropositivity results were seen in different seasons, suggesting that bites can occur throughout the year.

*B. burgdorferi* antibodies are developed relatively late in some patients, and indeed never in others. However, since several serum samples were available from some patients, a few cases of acute (subclinical or clinical) infection (presence of IgM and rising or falling IgG antibody titres) were clearly identified. The small number detected may be explained in that all patients presenting with fever symptoms were treated with antibiotics in accordance with the policy for the prevention and control of tick-borne diseases designed by the Servicio Territorial de Sanidad y Bienestar Social de la Junta de Castilla y León (the regional health service). Thus the true number of infections might have been masked by this early treatment [35]. Indeed, some authors have now clearly indicated that the early administration of antibiotics can postpone or inhibit the development of a response to the pathogen [36,37]. The present results may support these findings. Though some authors indicate that prophylactic antibiotic treatment should be administered after a tick bite others suggest it is not indicated given the low incidence of eventual disease [13,38,39]. Nevertheless, since the transmission of *B. burgdorferi* never occurs within the first 24 h of tick attachment (indeed, 48 h may be required), removing ticks as soon as possible is a good prevention method. Sometimes bites from several ticks are needed before antibodies to *B. burgdorferi* become detectable.

In Europe, *B. burgdorferi* is usually transmitted to humans via the tick* I. ricinus*, and mainly by its nymphs [2,3,40,41,42,43]. These are more active from spring to autumn and need 85% humidity in order to thrive. Tick species other than *I. ricinus* were, however, identified on the patients in the present work, including *D. marginatus*, *D. reticulatus*, *R. sanguineus* and *R. turanicus.*

PCR detected *B. burgdorferi* DNA in *D. reticulatus* males. *Dermacentor* spp. play an important role in the transmission of tick-borne pathogens that is not well recognized in Europe [44,45]. The latter authors reported the first epidemiological characterizations of pathogens spread via *Dermacentor* spp. in France. In the present work, *B. burgdorferi* DNA was detected in a *D. reticulatus* male. Other authors suggest* D. reticulatus* to have expanded its area of distribution [46]. Certainly, its growing presence in Serbia was reported [47]. Further experiments are needed to determine to the vector competency of this pathogen.

In summary, it is important to increase awareness of *B. burgdorferi* infection, and that better surveillance should be implemented. Further studies are planned, including studies using more antigens (like other serotypes and species of the *B. burgorferi* sensu stricto, *B. afzelii*, *B. garinii*, to avoid the loss of positive samples), more epidemiological studies and analyses of suspected clinical cases, to try to determine the true importance of *B. burgdorferi* infection.
